# The Effectiveness of Four Quadrivalent, Inactivated Influenza Vaccines Administered Alone or in Combination with Pneumococcal and/or SARS-CoV-2 Vaccines: A Population-Wide Cohort Study

**DOI:** 10.3390/vaccines13030309

**Published:** 2025-03-13

**Authors:** Cecilia Acuti Martellucci, Annalisa Rosso, Enrico Zauli, Alessandro Bianconi, Matteo Fiore, Graziella Soldato, Patrizia Marani Toro, Marco De Benedictis, Graziano Di Marco, Roberto Carota, Rossano Di Luzio, Maria Elena Flacco, Lamberto Manzoli

**Affiliations:** 1 Department of Medical and Surgical Sciences, University of Bologna, 40100 Bologna, Italy; c.acutimartellucci@unibo.it (C.A.M.); alessandro.bianconi4@studio.unibo.it (A.B.); matteo.fiore7@studio.unibo.it (M.F.); 2 Department of Environmental and Prevention Sciences, University of Ferrara, 44121 Ferrara, Italy; annalisa.rosso@unife.it (A.R.); mariaelena.flacco@unife.it (M.E.F.); 3 Department of Translational Medicine, University of Ferrara, 44121 Ferrara, Italy; enricozauli8@gmail.com; 4 Local Health Unit of Pescara, 65124 Pescara, Italy; graziella.soldato@ausl.pe.it (G.S.); patrizia.maranitoro@ausl.pe.it (P.M.T.); marco.debenedictis@ausl.pe.it (M.D.B.); graziano.dimarco@ausl.pe.it (G.D.M.); roberto.carota@ausl.pe.it (R.C.); rossano.diluzio@ausl.pe.it (R.D.L.)

**Keywords:** influenza, *Streptococcus pneumoniae*, SARS-CoV-2, vaccine effectiveness, cohort study, Italy

## Abstract

**Background**: Several influenza vaccine formulations are available, including adjuvanted, high-dose, trivalent, and quadrivalent vaccines, and direct, comparative evidence on the relative effectiveness is limited. Real-life data on the potential impact of the co-administration of pneumococcal and/or SARS-CoV-2 vaccinations are also very scarce. During the 2023–2024 influenza season, we carried out a retrospective cohort study on the entire elderly population of the Pescara province, Italy, in order to evaluate the effectiveness of the quadrivalent influenza vaccine, offered alone or in combination with other recommended vaccinations. **Methods**: All the immunization, demographic, co-payment, and hospitalization data were extracted from the official National Healthcare System, and the follow-up lasted from October 2023 to September 2024. The outcomes were all-cause mortality and hospital admissions for influenza and/or pneumonia. All the Cox models were adjusted (or stratified) for gender, age, hypertension, diabetes, COPD, CVD, renal disorders, cancer, and previous SARS-CoV-2 infection. **Results**: Overall, 43.9% of the population aged ≥60 years received an influenza vaccine (n = 46,355/105,527). A total of 3188 (3.0%) and 1047 (1.0%) individuals died of any cause or were hospitalized for influenza and/or pneumonia, respectively. During the follow-up, compared with the unvaccinated, those who received an influenza vaccine showed almost half the likelihood of death (adjusted HR: 0.52; 95%CI: 0.49–0.56) and hospitalization (aHR: 0.55; 95%CI: 0.48–0.62), regardless of the gender and age group. As compared with sole influenza immunization, the co-administration of a pneumococcal or COVID-19 vaccine was associated with a significantly lower risk of both outcomes. No substantial differences were observed by influenza vaccine formulation (MF59 adjuvanted; non-adjuvanted, standard dose; non-adjuvanted, high dose), with the exception of a greater mortality reduction for the MF59-adjuvanted vaccine as compared with the high-dose formulation. **Conclusions**: During the influenza season 2023–2024, all the influenza vaccines were largely effective among the elderly, with no substantial differences by formulation, age, or gender. However, the co-administration of a pneumococcal and/or SARS-CoV-2 vaccine further reduced the risk of both death and hospitalization. Specific, head-to-head randomized trials are required to confirm both findings.

## 1. Introduction

Seasonal influenza is a leading cause of infectious respiratory disease worldwide, causing 3 to 5 million cases of severe illness and 650,000 deaths each year [1,2], unevenly distributed among different population groups but largely prevailing among the elderly, who account for >90% of influenza-related deaths [3]. In this scenario, annual influenza vaccination has long been recognized as a pivotal tool to prevent influenza-like illness and mortality, particularly among high-risk individuals [4].

In Europe, following the recommendations of the European Medicines Agency Committee for Human Medicinal Products (CHMP), several vaccine formulations are available and updated each year [5], based upon the prevalence and characteristics of the circulating viruses [6]. As such, the current market is highly differentiated [7], with licensed trivalent and quadrivalent vaccines, containing two influenza A virus strains and either one (trivalent) or two (quadrivalent) influenza B virus strains [8]. Both vaccines are available in the MF59-adjuvanted or non-adjuvanted formulations and developed either at standard dose (containing 15 µg hemagglutinin antigen per strain) or at high dose (60 µg antigen per strain) [6]. The MF59-adjuvanted vaccines and non-adjuvanted high-dose formulations were specifically designed for the elderly [3], as the available evidence suggests that standard-dose vaccines can elicit only suboptimal protection among adults ≥65 years of age [3].

In this highly fragmented scenario, the available literature is dominated by randomized trials designed to assess the efficacy of individual vaccines, while direct, comparative evidence on the efficacy/effectiveness of the available vaccines is still limited [8]. This poses challenges to evidence-based decisions on the preferential use of one vaccine over another, mainly among high-risk individuals [9]. In particular, there is still inconclusive evidence on the comparative effectiveness of adjuvanted or high-dose quadrivalent vaccines (which were introduced in Europe from the season 2021/2022 [10]) over the standard-dose quadrivalent formulations [3,6,7,9].

In addition, it is well known that elderly individuals infected with influenza are more likely to harbor a secondary infection from *S. pneumoniae* [11] and to develop a pneumococcal disease, mainly pneumonia, which can in turn complicate influenza and severely contribute to morbidity and mortality [12]. Also, co-infection with *S. pneumoniae* has been reported in patients with COVID-19, in particular among elderly and high-risk individuals [13]. Therefore, given the overlapping target populations, a concomitant administration of pneumococcal [14,15] and SARS-CoV-2 vaccines is currently recommended [16]. Notably, previous randomized trials suggested a slightly lower seroresponse to pneumococcal immunization when administered in combination with influenza vaccination than that with pneumococcal vaccination alone [11], while other studies reported a comparable immunological response between the single and the combined schedules [11,12]. However, the available evidence is mostly based upon randomized trials, and real-life data from unselected populations are still limited and almost entirely based upon trivalent influenza vaccines [17,18].

In the context of a large, population-based vaccination program, the present study aims to evaluate the effectiveness of quadrivalent influenza vaccines (available as standard dose, high dose, or adjuvanted), offered alone or in combination with pneumococcal and/or anti-SARS-CoV-2 to all the residents aged ≥60 years of an Italian Province, in preventing all-cause mortality and hospital admissions for influenza and/or pneumonia.

## 2. Materials and Methods

### 2.1. Study Design and Data Source

This retrospective cohort study included all the permanent or temporary (domiciled) residents in the province of Pescara, Italy, on 10 October 2023 (the start of the influenza vaccination campaign), aged ≥60 years. We chose this age threshold to be consistent with the Italian Government [19], which identified subjects aged ≥60 years as priority targets for immunization. We used the same methodology adopted in previous studies in the same province on the effectiveness and safety of influenza [20] and SARS-CoV-2 vaccines [21,22,23] and extracted all the information from the following official National Healthcare System databases (Local Health Unit—LHU) that is collected routinely and used by the Italian Institute of Health [24]:

- Influenza and SARS-CoV-2 vaccinations (from 1 January 2021 to 10 May 2024, although 99.2% of the subjects were vaccinated before 1 March, 2024);
- Pneumococcal vaccinations (from 1 January 2016 to 10 May 2024);
- Demographic (Italian “Anagrafica”, up to 30 September 2024);
- Hospital admission discharge abstracts (Italian “SDO”, from 1 January 2014 to 30 September 2024).

To adjust for some of the main potential confounders of the association between the outcomes and vaccination status [25], we used (a) the above-mentioned hospital discharge abstracts and (b) the co-pay exemption database (Italian “Esenzioni Ticket”) from the last ten years to extract the following risk factors and comorbidities for each resident: hypertension (401.xx–405.xx); major cardiovascular or cerebrovascular diseases—CVD (410.xx–412.xx; 414.xx–415.xx; 428.xx or 433.xx–436.xx); diabetes (ICD-9-cm codes in any diagnosis field—250.xx); chronic obstructive pulmonary disease—COPD (491.xx–493.xx); kidney diseases (580.xx–589.xx); and cancer (140.xx–172.xx or 174.xx–208.xx). We also used the dataset of SARS-CoV-2 PCR or rapid antigenic tests (up to 10 October 2023), in order to adjust the analyses for previous SARS-CoV-2 infection. All the information from the above datasets was merged through an encrypted fiscal code.

### 2.2. Outcomes

The outcomes were all-cause mortality and hospital admissions for influenza and/or pneumonia (ICD-9-CM codes 480.0 through 487.8 in any diagnosis field [26,27,28]), recorded from 24 October 2023 (14 days after the start of follow-up) up to 30 September 2024. It was not possible to evaluate influenza-related mortality because the information in the LHU Death Registry is not updated. Nevertheless, the two outcomes were adopted also in view of their conventional use by randomized controlled trials testing the efficacy of the influenza and pneumococcal vaccines [9,29].

### 2.3. Exposure—Vaccinations

Following the recommendations of the Italian National vaccination plan [30] and the specific indications by the Italian Drug Agency (AIFA [31]), influenza, pneumococcal, and SARS-CoV-2 vaccinations are offered free of charge to all the Italian population.

Due to specific financial agreements between the Abruzzo Region and the producers, during the 2023–2024 influenza season, the following influenza vaccines were administered by the Local Health Unit of Pescara, free of charge, to all adults:

(a) Surface antigen, MF59-adjuvanted, Fluad Tetra (^®^Seqirus);
(b) Surface antigen, Flucelvax Tetra (^®^Seqirus);
(c) Split-virion, high-dose, Efluelda Tetra (^®^Sanofi, recommended only ≥60 years of age);
(d) Surface antigen (subunit), Influvac S Tetra (^®^Viatris).

All the vaccines were inactivated, administered in a single intramuscular dose of 0.5 mL, and quadrivalent: containing four hemagglutinin (HA) strains recommended by the World Health Organization (WHO) and European Union for the 2023–2024 Northern Hemisphere influenza season: A/Victoria/4897/2022 (H1N1) pdm09-like virus; A/Darwin/9/2021 (H3N2)-like virus; B/Austria/1359417/2021 (B/Victoria lineage)-like virus; B/Phuket/3073/2013 (B/Yamagata lineage)-like virus [32].

The following SARS-CoV-2 (1) and pneumococcal (2) vaccines were also administered during the 2023–2024 season:

1. mRNA-1273 (^®^Moderna), BNT162b2 (^®^Pfizer), or NVX-CoV2373 (^®^Novovax). A single dose was sufficient to be considered vaccinated against COVID-19.
2. Pneumococcal conjugate vaccines 15-valent (PCV15; ^®^Merck), 20-valent (PCV20; ^®^Pfizer), or polysaccharide vaccine (PPSV23; ^®^Merck). All the pneumococcal vaccinations administered before the 2023/2024 season were also retrieved, up to 1 Jan 2016 (which includes 97% of all vaccinations).

For each vaccine, individuals were considered as “vaccinated” only if they received the vaccine ≥14 days before the outcome.

### 2.4. Follow-Up

The start of the follow-up differed by vaccination status: 10 October 2023, for unvaccinated individuals; 14 days after the influenza immunization (to account for seroconversion time), for vaccinated subjects. The follow-up ended on the day of death or hospitalization (for the analyses on this outcome) or on 30 September 2024. As previously suggested [33], the follow-up was extended beyond the usual 6-month period in order to be able to evaluate the impact of the vaccination on long-term outcomes.

### 2.5. Data Analysis

The main analyses compared the rate of (a) death or (b) hospitalization of the subjects who received an influenza vaccine, alone or in combination with pneumococcal and/or SARS-CoV-2 vaccines, versus the unvaccinated. The adjusted hazard ratios (HRs) of both outcomes by immunization status were computed using a Cox proportional hazards analysis [23]. All the models were adjusted for gender, age, hypertension, diabetes, COPD, CVD, renal disorders, cancer, and previous SARS-CoV-2 infection (all included a priori). The mentioned comorbidities were extrapolated form the hospital admission and discharge abstracts (Italian “SDO”), considering all the hospitalizations that occurred from 1 January 2014 and up to 30 September 2023 for the unvaccinated or up to 14 days after the influenza vaccination date for the vaccinated. Nelson–Aalen cumulative hazard estimates were used to evaluate the validity of constant incidence ratios over the follow-up for all Cox models, and the Schoenfeld’s test was employed to evaluate the validity of the proportional hazards assumption [22]. The reference category was always the group of unvaccinated (for influenza), but the main analyses were repeated excluding this group, in order to directly compare the likelihood of death (or hospitalization) of the subjects who received different vaccines. All the analyses were also stratified by age (<75 y, ≥75 y), gender, influenza vaccine type, and whether pneumococcal and/or SARS-CoV-2 vaccines had also been received. Stata version 13.1 (Stata Corporation, College Station, TX, USA, 2014) was used for all the computations.

## 3. Results

### 3.1. Characteristics of the Sample

As shown in Figure S1, a total of 105,527 residents or individuals domiciled in the Pescara province, Italy, aged ≥60 years, were included in the analyses. As reported in Table 1, the mean age of the sample was 72.5 ± 9.6 years, and 45.4% were males. The prevalences of hypertension and diabetes mellitus were 30.4% and 11.2%, respectively. Overall, 43.9% of the population received an influenza vaccine (n = 46,355). Most of them received a MF59-adjuvanted influenza vaccine (71.7%; n = 33,248); 74.5% were immunized only against influenza (n = 34,549); 7.4% received both influenza and pneumococcal vaccines; 15.7% were vaccinated against both influenza and COVID-19; and 2.4% received all three vaccinations. The vaccinated individuals were significantly older, and their prevalence of all risk factors and comorbidities was much higher than that for the unvaccinated (all *p* < 0.001; Table 1). The mean follow-up of this study was 333 ± 43 days.

### 3.2. All-Cause Death

A total of 3188 individuals died of any cause (3.02% of the population) during the follow-up (Table 2). Despite the age differential, the raw death rate was similar among the vaccinated (2.98%) and unvaccinated (3.07%), in both genders (Table S1). The overall mortality approached 0.0% in the group of the subjects who also received the pneumococcal vaccine and was highest among those who received the split-virion, high-dosage influenza vaccine (9.9%), which was specifically recommended for the oldest (the mean age of the individuals who received this vaccine was 85.6 years).

At multivariable analyses (Table 2), compared with the unvaccinated, the likelihood of death almost halved among the individuals who received an influenza vaccine (adjusted HR: 0.52; 95% confidence interval—CI: 0.49–0.56), and this difference was consistent across genders and age class (Table S1). The risk of death was significantly lower for all types of influenza vaccines, in combination with any other vaccine, with HRs approaching zero for those who received both influenza and pneumococcal vaccines. When the analyses were restricted to the vaccinated individuals, the co-administration of a COVID-19 vaccine was also associated with a significantly lower probability of death than that for the sole influenza immunization (adjusted HR: 0.71; 95% CI: 0.61–0.82). In contrast, the high-dose, split-virion vaccine showed a significantly higher mortality than the MF59-adjuvanted one, both among the subjects who were only immunized against influenza and among those who also received a SARS-CoV-2 vaccine (Table S2).

No substantial differences were observed among the other influenza vaccines, both in the overall sample and when the analyses were stratified by gender, age class, or the co-administration of other vaccines (Table 1, Tables S1 and S2).

### 3.3. Hospitalizations for Influenza or Pneumonia

During the follow-up, 1047 persons (0.99% of the population) were admitted to a hospital for influenza and/or pneumonia (Table 2). The crude hospitalization rate was similar for vaccinated versus unvaccinated individuals but significantly lower for individuals who also received a pneumococcal vaccine.

In multivariable analyses, similar to what was observed for all-cause mortality, the subjects who were immunized against influenza showed almost half the likelihood of hospitalization compared with the unvaccinated (adjusted HR: 0.55; 95% CI: 0.48–0.62), regardless of the gender and age group (Table S1). The risk of a hospital admission was significantly lower for all types of influenza vaccines, in combination with any other vaccine, with the only exception of the surface, subunit antigen B, likely due to the scarce number of admissions (Table 2). Again, no substantial differences were observed in the stratified analyses, restricted to the vaccinated individuals, across genders and age groups, but unlike mortality, varying the vaccine type or co-administering another vaccine did not significantly vary the risk of hospitalization (Table 2, Tables S1 and S2).

## 4. Discussion

The main findings from this retrospective cohort analysis of the whole elderly population of an Italian province are the following: (a) during influenza season 2023–2024, the subjects who received a quadrivalent, inactivated influenza vaccine, as compared with the unvaccinated, showed approximately half the risk of death or hospitalization due to influenza and/or pneumonia; (b) the individuals who also received a pneumococcal and/or COVID-19 vaccination showed a significantly lower risk of both outcomes than those who were vaccinated solely against influenza, with mortality rates approaching 0% among the recipients of a pneumococcal vaccine; (c) no significant differences were observed by gender, age-class, and type of influenza vaccine.

Although the literature on the efficacy of influenza vaccination is massive [8,33,34,35,36], limited evidence is available on the real-world effectiveness of inactivated, quadrivalent influenza vaccines to prevent hospitalizations [6,37,38,39,40,41,42]. Interestingly, while we used a traditional cohort design and extracted ICD-9-CM codes from discharge abstracts, the other published studies on the 2023–2024 season had a test-negative case–control design, with lab confirmation of the diagnoses [6,38,39,41]. Although the absence of a laboratory confirmation enhances the likelihood of healthy vaccinee bias and confounding by indication [43,44], the use of discharge abstracts allowed the inclusion of the entire population, to adjust for some major risk factors and comorbidities, and ultimately resulted in findings that were consistent with all previous studies, from other countries [38,39,40,41] or Italian regions [6,42], in which vaccine effectiveness ranged from 35% to 53%. Furthermore, the present findings are in line with the estimates from meta-analyses that considered different seasons and other vaccine types [4,34].

The evidence on the effectiveness of quadrivalent vaccines on mortality is also limited, based upon in-hospital data only, with no population-wide studies and follow-ups shorter than six months [39,45,46]. As mentioned, we included all deaths, the whole population, and a longer follow-up. However, the present results were consistent with the available meta-analyses of observational studies, [47,48], as well as with a previous study from the same influenza season [39]. In the same season, a small study on hospitalized patients from Spain reported a vaccine effectiveness as high as 79% against death/intensive unit admission [45]. With that regard, it should be considered that, despite the adjustment for confounders, all observational analyses, including the present, are likely to overestimate the vaccine effectiveness to prevent all-cause mortality, due to the frail selection bias and the above-mentioned healthy vaccinee bias [44,47].

A growing body of evidence, including two studies on quadrivalent vaccines [3,6], reported a higher effectiveness of enhanced influenza vaccines (recombinant, adjuvanted, or high-dose formulations) versus standard ones [4,7,9,43,49,50,51,52,53,54], and they have been suggested as a preferable option for older individuals [55,56]. In the present study, two quadrivalent enhanced vaccines were administered during the 2023–2024 influenza season and could be evaluated: a MF59-adjuvanted and a high-dose, split-virion vaccine. While both enhanced formulations were able to significantly and substantially reduce the likelihood of both death and hospitalization due to influenza and/or pneumonia among the recipients, as compared to the unvaccinated, no significant differences in vaccine effectiveness were observed between both enhanced formulations and the other standard vaccines that were distributed during the study period. In contrast, the MF59-adjuvanted vaccine showed a significantly higher effectiveness than the high-dose, non-adjuvanted vaccine in preventing both death and hospital admissions, whereas most previous studies found no significant differences in the clinical effectiveness of the commercially available enhanced formulations [6,7,49,53]. A potential explanation for this finding lies in the large discrepancy in the distribution of very old subjects (≥80 y) and comorbidities between the high-dose group and the others. In the present population, the recipients of the high-dose vaccine were 9 years older on average and showed significantly higher rates of comorbidities than those receiving the adjuvanted formulation: although our models were adjusted for both age and concomitant chronic conditions, it is likely that some residual confounding by indication remained, as suggested by a similar study conducted on quadrivalent inactivated vaccines [3]. Further studies are needed to draw more substantial conclusions on the potential benefits of enhanced quadrivalent influenza vaccines, hopefully using a head-to-head randomized design, as no such trials are currently available.

A recent meta-analysis found a higher vaccination efficacy among the elderly females, suggesting the need to generate sex-disaggregated data on vaccination outcomes in order to better understand the role of sex and possibly define more tailored vaccination strategies [57]. Indeed, we stratified all analyses by gender and age, but, in the present population, we found no significant differences by gender. It is worth noting, however, that the above meta-analysis was based upon RCT data, and the available observational evidence did not provide conclusive results [58]. With regard to age, we found the highest absolute risk reduction among individuals aged 75 years or more (a 3.1% and 0.8% decrease in deaths and hospitalizations, respectively), which is consistent with previous research [54,59] and reflects the higher background risk in this population [60].

We observed a substantial and significant reduction in the risk of both death and hospital admission in the sub-sample of individuals receiving influenza and pneumococcal or SARS-CoV-2 vaccines versus influenza alone. This incremental effectiveness is consistent with the available literature, mostly based upon RCTs, in which the reported decrease in hospitalization rates among the elderly receiving a combined (versus sequential) pneumococcal and influenza vaccination ranged from 46% to 78% [61,62,63,64], while the risk of all-cause death was reduced from a minimum of 19% to a maximum of 57% [17,61,62,65,66]. Such large fluctuations may be due to the characteristics of the population evaluated [17], the degree of matching between vaccine and circulating influenza viruses [65], the accuracy of the outcome assessment [65], and/or the time at which the studies were conducted, as vaccine performance might be underestimated during periods of higher influenza activity [17]. Importantly, most of the previous studies (with the exception of a small evaluation performed among individuals living in nursing homes in Turkey [64]) were performed before the 2021–2022 influenza season, when the trivalent formulation was still available and recommended, while the present analysis is, to our knowledge, the first performed in the context of a vaccination campaign entirely based upon quadrivalent vaccines [10].

To our knowledge, this is the first study to evaluate the potential impact of a co-administration of influenza and pneumococcal or COVID-19 vaccinations in a large, unselected sample of elderly individuals. The main strengths of this study are the large sample size, the inclusion of the entire population of a province, the use of official, routinely collected electronic health databases, and the adjustment for multiple comorbidities. However, this study has some limitations that must be considered in interpreting the results. First, the reliance on hospital ICD9-CM codes to identify influenza and pneumonia cases may capture only the more severe cases of influenza illness, which may lead to an overestimation of the overall vaccine effectiveness. Second, as previously mentioned, all observational studies on vaccines are prone to confounding by indication, which we tried to reduce through the adjustment for several risk factors and comorbidities. Third, we could not assess influenza-specific mortality, as the death-causes dataset becomes available with an average delay of three years, and we also could not ensure the capture of all influenza infections among hospitalized people, as there is no requirement to systematically test for influenza or other infectious agents. Still, the investigated outcomes correspond to those conventionally used in RCTs [9,29] and allow the formulation of hypotheses on the differences (beyond the circulating viral types) between efficacy in experimental conditions and effectiveness in a large, unselected population. Finally, during the 2023–2024 influenza season, only quadrivalent vaccines were administered in the Province of Pescara. Thus, it was impossible to obtain data on the potential advantage of quadrivalent versus trivalent vaccines, which has been questioned due to the virtual disappearance of influenza B/Yamagata detections in recent years [67].

## 5. Conclusions

In the elderly population of an Italian province, during the influenza season 2023–2024, all the administered quadrivalent, inactivated influenza vaccines were able to effect a substantial and significant decrease in the risk of death or hospitalization due to influenza and/or pneumonia. The co-administration of a pneumococcal and/or SARS-CoV-2 vaccine further reduced the risk of both outcomes, while no significant differences were observed by gender, age-class, and between enhanced and standard vaccines. Head-to-head, randomized trials are required to confirm the specific benefits of co-administration with other vaccines and to compare the different types of quadrivalent influenza vaccine formulations.

## Figures and Tables

**Table 1:** Characteristics of the sample: overall and by influenza vaccination status.

Variables	Overall	Vaccinated	Unvaccinated	
	(n = 105,527)	(n = 46,355; 43.9%)	(n = 59,172; 56.1%)	*p* ^A^
Male gender, % (n)	45.4 (47,926)	45.2 (20,927)	45.6 (26,999)	0.118
Age				
- Mean age in years (SD)	72.5 (9.6)	76.0 (9.0)	69.7 (9.1)	<0.001
- Age < 75 years, % (n)	58.7 (61,932)	43.2 (20,043)	29.2 (17,283)	<0.001
- Age ≥ 75 years, % (n)	41.3 (43,595)	56.8 (26,312)	70.8 (41,889)	<0.001
Risk factors and comorbidities ^B^				
- Hypertension, % (n)	30.4 (32,074)	41.8 (19,377)	21.5 (12,697)	<0.001
- Previous CVD, % (n)	16.3 (17,244)	22.6 (10,477)	11.4 (6767)	<0.001
- Diabetes, % (n)	11.2 (11,782)	15.5 (7165)	7.8 (4617)	<0.001
- COPD, % (n)	5.5 (5775)	7.9 (3669)	3.6 (2106)	<0.001
- Kidney disease, % (n)	3.8 (3974)	5.3 (2459)	2.6 (1515)	<0.001
- Past cancer diagnosis, % (n)	10.9 (11,541)	14.0 (6497)	8.5 (5044)	<0.001
- Past SARS-CoV-2 infection, % (n)	33.9 (35,817)	35.8 (16,599)	32.5 (19,218)	<0.001
Vaccination status ^C^, % (n)				
- No vaccination	56.1 (59,172)	--	100.0 (59,172)	--
- Flu vaccine only	32.7 (34,549)	74.5 (34,549)	--	--
- Flu and pneumococcal vaccine	3.2 (3431)	7.4 (3431)	--	--
- Flu and SARS-CoV-2 vaccine	6.9 (7268)	15.7 (7268)	--	--
- All 3 vaccines	1.1 (1107)	2.4 (1107)	--	--
Vaccine type ^D^, % (n)				
- MF59-adjuvanted ^E^	31.5 (33,248)	71.7 (33,248)	--	--
- Non-adjuvanted, standard dose 1 ^F^	7.0 (7372)	15.9 (7372)	--	--
- Non-adjuvanted, high dose ^G^	4.0 (4242)	9.2 (4242)	--	--
- Non-adjuvanted, standard dose 2 ^H^	1.4 (1493)	3.2 (1493)	--	--
Mean days of follow-up (SD) ^I^	333 (43)	310 (38)	350 (38)	<0.001

SD: standard deviation. ^A^ Chi-squared test for categorical variables; *t*-test for continuous ones. ^B^ Details are available in the Materials and Methods Section. Only the risk factors and comorbidities that were identified before vaccination (or the start of follow-up, for the unvaccinated subjects) were included. ^C^ Individuals who received at least one dose of influenza and/or pneumococcal and/or SARS-CoV-2 vaccine from 10 October 2023 up to 10 May 2024 (99.2% of the subjects were vaccinated before 1 March 2024) and/or at least one dose of pneumococcal vaccine from 1 January 2016 to 10 May 2024. For each vaccine, individuals were considered as “vaccinated” only if they received the vaccine ≥14 days before the outcome. ^D^ All the vaccines were quadrivalent, inactivated influenza vaccines, containing four hemagglutinin (HA) strains recommended by the World Health Organization (WHO) and the EU for the 2023–2024 Northern Hemisphere influenza season: A/Victoria/4897/2022 (H1N1) pdm09-like virus; A/Darwin/9/2021 (H3N2)-like virus; B/Austria/1359417/2021 (B/Victoria lineage)-like virus; B/Phuket/3073/2013 (B/Yamagata lineage)-like virus. ^E^ Fluad Tetra ^®^(Seqirus). ^F^ Flucelvax Tetra ^®^(Seqirus). ^G^ Efluelda Tetra ^®^(Sanofi). ^H^ Influvac S Tetra ^®^(Viatris). ^I^ The follow-up ended on the date of death (or hospitalization, for the analyses of this outcome) or on 30 September 2024. The start of follow-up varied across vaccine categories: (a) two weeks after influenza vaccination; (b) 10 October 2023 for the unvaccinated subjects.

**Table 2:** Outcomes of this study: overall and by vaccination status.

	Death			Hospitalization		
	% (n)	Adjusted HR(95% CI) ^A^	*p* ^A^	% (n)	Adjusted HR(95% CI) ^A^	*p* ^A^
Overall sample (n = 105,527)	3.02 (3188)	--	--	0.99 (1047)	--	--
Influenza vaccination status ^B^						
- Unvaccinated	2.98 (1765)	1 (Ref. cat.)	--	0.96 (567)	1 (Ref. cat)	--
- Vaccinated, overall	3.07 (1423)	0.52 (0.49–0.56)	<0.001	1.04 (480)	0.55 (0.48–0.62)	<0.001
- Flu vaccine only	3.51 (1213)	0.60 (0.56–0.65)	<0.001	1.10 (381)	0.59 (0.51–0.67)	<0.001
- Flu and pneumococcal vaccine	0.0 (0)	0.00 (NE)	0.999	0.52 (18)	0.33 (0.20–0.52)	<0.001
- Flu + SARS-CoV-2 vaccine	2.88 (209)	0.43 (0.37–0.49)	<0.001	0.99 (72)	0.48 (0.38–0.62)	<0.001
- All 3 vaccines	0.09 (1)	0.02 (0.00–0.11)	<0.001	0.81 (9)	0.41 (0.21–0.80)	0.009
Influenza vaccine type ^C^, % (n)						
- No vaccine	2.98 (1765)	1 (Ref. cat.)	--	1.00 (331)	1 (Ref. cat.)	--
- MF59-adjuvanted ^D^	2.77 (920)	0.48 (0.45–0.52)	<0.001	1.00 (331)	0.53 (0.46–0.60)	<0.001
- Non-adjuvanted, standard dose 1 ^E^	0.92 (68)	0.47 (0.37–0.59)	<0.001	0.43 (32)	0.53 (0.37–0.76)	0.001
- Non-adjuvanted, high dose ^F^	9.88 (419)	0.66 (0.59–0.74)	<0.001	2.50 (106)	0.62 (0.50–0.77)	<0.001
- Non-adjuvanted, standard dose 2 ^G^	1.07 (16)	0.54 (0.33–0.88)	0.013	0.74 (11)	0.89 (0.49–1.62)	0.705
Vaccinated only (n = 46,355)		Adjusted HR(95% CI) ^H^	*p* ^H^		Adjusted HR(95% CI) ^H^	*p* ^H^
Influenza vaccination status ^B^						
- Flu vaccine only		1 (Ref. cat.)	--		1 (Ref. cat.)	--
- Flu and pneumococcal vaccine		0.00 (NE)	0.999		0.53 (0.33–0.86)	0.009
- Flu and SARS-CoV-2 vaccine		0.71 (0.61–0.82)	<0.001		0.82 (0.64–1.06)	0.127
- All 3 vaccines		0.03 (0.00–0.19)	<0.001		0.68 (0.35–1.33)	0.261
Influenza vaccine type C, % (n)						
- MF59-adjuvanted ^D^		1 (Ref. cat.)	--		1 (Ref. cat.)	--
- Non-adjuvanted, standard dose 1 ^E^		0.98 (0.76–1.26)	0.872		0.91 (0.63–1.32)	0.620
- Non-adjuvanted, high dose ^F^		1.34 (1.18–1.51)	<0.001		1.31 (1.03–1.65)	0.026
- Non-adjuvanted, standard dose 2 ^G^		1.11 (0.68–1.83)	0.673		1.51 (0.82–2.76)	0.187

HR: hazard ratio. CI: confidence interval. NE: not estimable. Ref. Cat.: reference category. ^A^ Based on Cox proportional hazards models, adjusting for age; gender; hypertension; diabetes; previous cardiovascular, kidney, and/or chronic obstructive pulmonary diseases; cancer; and previous SARS-CoV-2 infection. The unvaccinated subjects are the reference group for all HRs. ^B^ Please see Table 1, footnote C. ^C^ All the vaccines were quadrivalent, inactivated influenza vaccines, containing four hemagglutinin (HA) strains recommended by the World Health Organization (WHO) and the EU for the 2023–2024 Northern Hemisphere influenza season: A/Victoria/4897/2022 (H1N1) pdm09-like virus; A/Darwin/9/2021 (H3N2)-like virus; B/Austria/1359417/2021 (B/Victoria lineage)-like virus; B/Phuket/3073/2013 (B/Yamagata lineage)-like virus. ^D^ Fluad Tetra ^®^(Seqirus). ^E^ Flucelvax Tetra ^®^(Seqirus). ^F^ Efluelda Tetra ^®^(Sanofi). ^G^ Influvac S Tetra ^®^(Viatris). ^H^ Based on Cox proportional hazards models, restricted to vaccinated subjects, adjusting for age; gender; hypertension; diabetes; previous cardiovascular, kidney, and/or chronic obstructive pulmonary diseases; cancer; and previous SARS-CoV-2 infection. The reference category was the group of individuals who received the surface antigen, MF59-adjuvanted vaccine.

## Data Availability

The data presented in this study are available upon reasonable request from the corresponding author.

## Supplementary Materials

The following supporting information can be downloaded at https://www.mdpi.com/article/10.3390/vaccines13030309/s1: Figure S1: Flowchart in line with the STROBE (Strengthening the Reporting of Observational Studies in Epidemiology) statement (http://www.strobestatement.org); Table S1: Outcomes of this study by vaccination status stratified by gender and age class; Table S2: Outcomes stratified by vaccination status and influenza vaccine type (vaccinated individuals only).
