# Supplementary material for: The Effectiveness of Four Quadrivalent, Inactivated Influenza Vaccines Administered Alone or in Combination with Pneumococcal and/or SARS-CoV-2 Vaccines: A Population-Wide Cohort Study
Source: Vaccines (Basel). 2025 Mar 13;13(3):309. doi: 10.3390/vaccines13030309 (PMC11946095; doi:10.3390/vaccines13030309)
Supplement: Supplementary file 1 [file vaccines-13-00309-s001.zip › vaccines-3506070-supplementary.pdf]

Figure S1. Study flowchart in line with the STROBE (Strengthening the Reporting of Observational Studies in Epidemiology) statement (http://www.strobestatement.org).

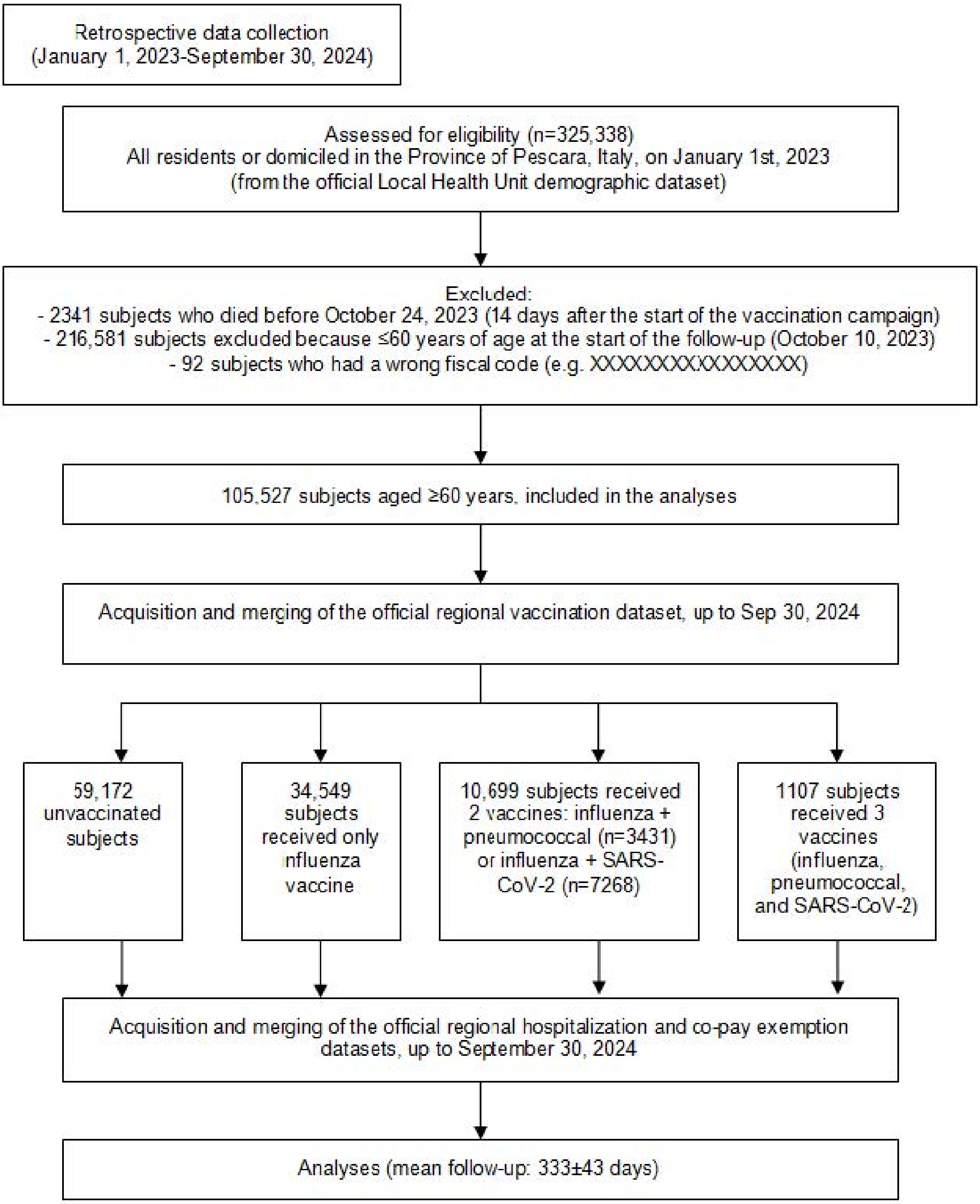

Table S1. Outcomes of the study by vaccination status, stratified by gender and age class.

	Death			Hospitalization		
	% (n)	Adjusted HR (95% CI) [A]	p [A]	% (n)	Adjusted HR (95% CI) [A]	p [A]
Females (n=57,601)	2.89 (1664)	--	--	0.85 (491)	--	--
- Unvaccinated	2.87 (902)	1 (Ref. cat.)	--	0.87 (281)	1 (Ref. cat.)	--
- Vaccinated, overall [B]	3.00 (762)	0.60 (0.54-0.66)	<0.001	0.83 (210)	0.51 (0.43-0.62)	<0.001
- MF59-adjuvanted [C]	2.60 (470)	0.55 (0.50-0.62)	<0.001	0.79 (143)	0.51 (0.41-0.62)	<0.001
- Non-adjuvanted, standard dose 1 [D]	0.80 (32)	0.50 (0.35-0.72)	<0.001	0.30 (12)	0.43 (0.24-0.77)	0.004
- Non-adjuvanted, high-dose [E]	9.87 (252)	0.74 (0.64-0.86)	<0.001	1.96 (50)	0.55 (0.40-0.75)	<0.001
- Non-adjuvanted, standard dose 2 [F]	0.99 (8)	0.56 (0.28-1.12)	0.099	0.62 (5)	0.81 (0.33-1.96)	0.642
Males (n=47,926)	3.18 (1524)	--	--	1.16 (556)	--	--
- Unvaccinated	3.20 (863)	1 (Ref. cat.)	--	1.06 (286)	1 (Ref. cat.)	--
- Vaccinated, overall [B]	3.16 (661)	0.45 (0.41-0.50)	<0.001	1.29 (270)	0.58 (0.49-0.69)	<0.001
- MF59-adjuvanted [C]	2.97 (450)	0.43 (0.38-0.48)	<0.001	1.24 (188)	0.55 (0.45-0.66)	<0.001
- Non-adjuvanted, standard dose 1 [D]	1.06 (36)	0.43 (0.31-0.60)	<0.001	0.59 (20)	0.63 (0.40-0.99)	0.043
- Non-adjuvanted, high-dose [E]	9.89 (167)	0.57 (0.48-0.68)	<0.001	3.32 (56)	0.69 (0.51-0.93)	0.015
- Non-adjuvanted, standard dose 2 [F]	1.17 (8)	0.51 (0.25-1.02)	<0.001	0.88 (6)	0.97 (0.43-2.17)	0.936
	% (n)	Adjusted HR (95% CI) [G]	p [G]	% (n)	Adjusted HR (95% CI) [G]	p [G]
Age<75 years (n=61,932)	0.80 (494)	--	--	0.40 (245)	--	--
- Unvaccinated	0.88 (369)	1 (Ref. cat.)	--	0.39 (163)	1 (Ref. cat.)	--
- Vaccinated, overall [B]	0.62 (125)	0.50 (0.41-0.62)	<0.001	0.41 (82)	0.62 (0.47-0.82)	0.001
- MF59-adjuvanted [C]	0.69 (86)	0.52 (0.41-0.67)	<0.001	0.40 (49)	0.56 (0.40-0.78)	0.001
- Non-adjuvanted, standard dose 1 [D]	0.38 (23)	0.37 (0.24-0.56)	<0.001	0.33 (20)	0.62 (0.39-0.98)	0.043
- Non-adjuvanted, high-dose [E]	2.58 (11)	1.48 (0.80-2.72)	0.208	1.88 (8)	1.81 (0.87-3.73)	0.111
- Non-adjuvanted, standard dose 2 [F]	0.42 (5)	0.40 (0.16-0.96)	0.040	0.42 (5)	0.70 (0.29-1.71)	0.433
Age≥75 years (n=43,595)	6.18 (2694)	--	--	1.84 (802)	--	--
- Unvaccinated	8.08 (1396)	1 (Ref. cat.)	--	2.34 (404)	1 (Ref. cat.)	--
- Vaccinated, overall [B]	4.93 (1298)	0.57 (0.52-0.61)	<0.001	1.51 (398)	0.55 (0.48-0.63)	<0.001
- MF59-adjuvanted [C]	4.00 (834)	0.47 (0.43-0.51)	<0.001	1.35 (282)	0.50 (0.43-0.59)	<0.001
- Non-adjuvanted, standard dose 1 [D]	3.32 (45)	0.43 (0.32-0.57)	<0.001	0.88 (12)	0.35 (0.20-0.63)	<0.001
- Non-adjuvanted, high-dose [E]	10.7 (408)	1.09 (0.97-1.22)	0.140	2.57 (98)	0.80 (0.64-1.00)	0.050
- Non-adjuvanted, standard dose 2 [F]	3.79 (11)	0.48 (0.26-0.86)	0.014	2.07 (6)	0.82 (0.36-1.83)	0.623

HR: Hazard Ratio. CI: Confidence Interval. Ref. Cat.: Reference category. [A] Based on Cox proportional hazards models, adjusting for age, hypertension, diabetes, previous cardiovascular, kidney, and/or chronic obstructive pulmonary diseases, cancer, and previous SARS-CoV-2 infection. The unvaccinated subjects are the reference group for all HRs. [B] Please see Table 1 footnote C. [C] All vaccines were quadrivalent, inactivated influenza vaccines, containing four hemagglutinin (HA) strains recommended by the World Health Organization (WHO) and EU for the 2023–2024 Northern Hemisphere influenza season: A/Victoria/4897/2022 (H1N1) pdm09-like virus; A/Darwin/9/2021 (H3N2)-like virus; B/Austria/1359417/2021 (B/Victoria lineage)-like virus; B/Phuket/3073/2013 (B/Yamagata lineage)-like virus. [D] Fluad Tetra ® (Seqirus). [E] Flucelvax Tetra ® (Seqirus). [F] Efluelda Tetra ® (Sanofi). [G] Influvac S Tetra ® (Viatris). [G] Based on Cox proportional hazards models, adjusting for gender, hypertension, diabetes, previous cardiovascular, kidney, and/or chronic obstructive pulmonary diseases, cancer, and previous SARS-CoV-2 infection. The unvaccinated subjects are the reference group for all HRs.

Table S2. Outcomes stratified by vaccination status and influenza vaccine type (vaccinated individuals only).

Vaccination status [B]	Overall	Death			Hospitalization		
	% (n)	% (n)	Adjusted HR (95% CI) [A]	p [A]	% (n)	Adjusted HR (95% CI) [A]	p [A]
Flu vaccine only (N=34,549)							
- MF59-adjuvanted [C]	71.3 (24,632)	3.3 (805)	1 (Ref. cat.)	--	1.1 (271)	1 (Ref. cat.)	--
- Non-adjuvanted, standard dose 1 [D]	16.5 (5692)	1.0 (57)	0.95 (0.72-1.24)	0.689	0.4 (21)	0.72 (0.45-1.13)	0.151
- Non-adjuvanted, high-dose [E]	8.7 (2993)	11.2 (336)	1.27 (1.11-1.46)	<0.001	2.7 (80)	1.22 (0.94-1.60)	0.140
- Non-adjuvanted, standard dose 2 [F]	3.6 (1232)	1.2 (15)	1.06 (0.64-1.77)	0.823	0.7 (9)	1.34 (0.68-2.62)	0.396
Flu and pneumococcal vaccine (N=3431)							
- MF59-adjuvanted [C]	75.3 (2585)	0.0 (0)	1 (Ref. cat.)	--	0.5 (12)	1 (Ref. cat.)	--
- Non-adjuvanted, standard dose 1 [D]	13.9 (477)	0.0 (0)	NE	--	0.8 (4)	2.70 (0.76-9.56)	0.124
- Non-adjuvanted, high-dose [E]	7.9 (272)	0.0 (0)	NE	--	0.7 (2)	0.86 (0.17-4.33)	0.857
- Non-adjuvanted, standard dose 2 [F]	2.8 (97)	0.0 (0)	NE	--	0.0 (0)	0.00 (NE)	0.999
Flu and SARS-CoV-2 vaccine (N=7268)							
- MF59-adjuvanted [C]	71.5 (5195)	2.2 (115)	1 (Ref. cat.)	--	0.8 (42)	1 (Ref. cat.)	--
- Non-adjuvanted, standard dose 1 [D]	14.7 (1071)	1.0 (11)	1.01 (0.54-1.90)	0.970	0.7 (7)	1.62 (0.71-3.69)	0.250
- Non-adjuvanted, high-dose [E]	11.7 (853)	9.6 (82)	2.27 (1.66-3.09)	<0.001	2.5 (21)	1.78 (1.00-3.14)	0.049
- Non-adjuvanted, standard dose 2 [F]	2.1 (149)	0.7 (1)	0.80 (0.11-5.78)	0.827	1.3 (2)	4.66 (1.08-20.1)	0.039
All 3 vaccines (N=1107)							
- MF59-adjuvanted [C]	75.5 (836)	0.0 (0)	1 (Ref. cat.)	--	0.7 (6)	1 (Ref. cat.)	--
- Non-adjuvanted, standard dose 1 [D]	11.9 (132)	0.0 (0)	NE	--	0.0 (0)	0.00 (NE)	0.990
- Non-adjuvanted, high-dose [E]	11.2 (124)	0.8 (1)	NE	--	2.4 (3)	3.10 (0.71-13.5)	0.131
- Non-adjuvanted, standard dose 2 [F]	1.4 (15)	0.0 (0)	NE	--	0.0 (0)	0.00 (NE)	0.999
Vaccine type	n (%)		Adjusted HR (95% CI) [A]	p [A]		Adjusted HR (95% CI) [A]	p [A]
MF59-adjuvanted [C] (N=33,248)							
- Flu vaccine only	74.1 (24,632)	3.3 (805)	1 (Ref. cat.)	--	1.1 (271)	1 (Ref. cat.)	--
- Flu and pneumococcal vaccine	7.8 (2585)	0.0 (0)	Excluded*	--	0.5 (12)	0.50 (0.28-0.89)	0.018
- Flu and SARS-CoV-2 vaccine	15.6 (5195)	2.2 (115)	0.61 (0.50-0.74)	<0.001	0.8 (42)	0.68 (0.49-0.94)	0.021
- All 3 vaccines	2.5 (836)	0.0 (0)	Excluded*	--	0.7 (6)	0.63 (0.28-1.42)	0.265
Non-adjuvanted, standard dose 1 [D] (N=7372)							
- Flu vaccine only	77.2 (5692)	1.0 (57)	1 (Ref. cat.)	--	0.4 (21)	1 (Ref. cat.)	--
- Flu and pneumococcal vaccine	6.5 (477)	0.0 (0)	Excluded*	--	0.8 (4)	2.21 (0.75-6.52)	0.150
- Flu and SARS-CoV-2 vaccine	14.5 (1071)	1.0 (11)	0.77 (0.40-1.48)	0.436	0.7 (7)	1.60 (0.67-3.81)	0.285
- All 3 vaccines	1.8 (132)	0.0 (0)	Excluded*	--	0.0 (0)	0.00 (NE)	0.999
Non-adjuvanted, high-dose [E] (N=4242)							
- Flu vaccine only	70.6 (2993)	11.2 (336)	1 (Ref. cat.)	--	2.7 (80)	1 (Ref. cat.)	--
- Flu and pneumococcal vaccine	6.4 (272)	0.0 (0)	Excluded*	--	0.7 (2)	0.26 (0.06-1.06)	0.060
- Flu and SARS-CoV-2 vaccine	20.1 (853)	9.6 (82)	0.86 (0.68-1.10)	0.232	2.5 (21)	1.00 (0.61-1.62)	0.986
- All 3 vaccines	2.9 (124)	0.81 (1)	Excluded*	--	2.4 (3)	0.91 (0.29-2.89)	0.872

Non-adjuvanted, standard dose 2 [F] (N=1493)							
- Flu vaccine only	82.5 (1232)	1.2 (15)	1 (Ref. cat.)	--	0.7 (9)	1 (Ref. cat.)	--
- Flu and pneumococcal vaccine	6.5 (97)	0.0 (0)	Excluded*	--	0.0 (0)	0.00 (NE)	0.999
- Flu and SARS-CoV-2 vaccine	10.0 (149)	0.7 (1)	0.70 (0.09-5.45)	0.734	1.3 (2)	1.87 (0.39-9.03)	0.437
- All 3 vaccines	1.0 (15)	0.0 (0)	Excluded*	--	0.0 (0)	0.00 (NE)	0.999

HR: Hazard Ratio. CI: Confidence Interval. NE: Not estimable. Ref. Cat.: Reference category. [A] Based on Cox proportional hazards models, adjusting for age, gender, hypertension, diabetes, previous cardiovascular, kidney, and/or chronic obstructive pulmonary diseases, cancer, and previous SARS-CoV-2 infection. [B] Please see Table 1 footnote C. [C, D, E, F] Please see Table 1 footnotes with identical letters. * The two groups were excluded from the analyses because of the very scarce number of deaths, and a direct comparison was made between the individuals who received influenza vaccine only (reference category) and those who received both influenza and SARS-CoV-2 vaccines.
